# Supplementary material for: MetaRibo-Seq measures translation in microbiomes
Source: Nat Commun. 2020 Jun 29;11:3268. doi: 10.1038/s41467-020-17081-z (PMC7324362; doi:10.1038/s41467-020-17081-z)
Supplement: Supplementary file 10 — Supplementary Data 7 [file 41467_2020_17081_MOESM10_ESM.zip › File2/Confidence_High_Taxonomy/85309_out.krona.html]

Javascript must be enabled to view this page.

members
magnitude
magnitudeUnassigned
count
unassigned
taxon
rank

85309\_out

13

2
13
superkingdom

1239
phylum
13

class
13
186801

order
13
186802

1898207
species
13

SRS013951\_contig\_number\_25790SRS014948\_contig\_number\_contig-100\_2278.128614SRS017916\_contig\_number\_10596SRS019496\_contig\_number\_contig-100\_31835.31836SRS046369\_contig\_number\_6352SRS049773\_contig\_number\_36471SRS055017\_contig\_number\_contig-100\_9026.9027SRS063518\_contig\_number\_25919SRS1041033\_contig\_number\_contig-100\_1218.110853SRS143417\_contig\_number\_1607SRS143895\_contig\_number\_4972SRS144537\_contig\_number\_24827SRS148196\_contig\_number\_35270
